# Taxonomic status of otter species in Nakai‐Nam Theun National Park, Lao PDR, based on DNA evidence

**DOI:** 10.1002/ece3.9601

**Published:** 2022-12-21

**Authors:** Camille N. Z. Coudrat, Wanlop Chutipong, Manakorn Sukmak, Supaphen Sripiboon, Worata Klinsawat

**Affiliations:** ^1^ Association Anoulak Nakai District Khammouan Province Laos; ^2^ Conservation Ecology Program, Pilot Plant Development and Training Institute King Mongkut's University of Technology Thonburi Bangkok Thailand; ^3^ Department of Farm Resources and Production Medicine, Faculty of Veterinary Medicine Kasetsart University Nakhon Pathom Thailand; ^4^ Department of Large Animal and Wildlife Clinical Sciences, Faculty of Veterinary Medicine Kasetsart University Nakhon Pathom Thailand; ^5^ Conservation Ecology Program, School of Bioresources and Technology King Mongkut's University of Technology Thonburi Bangkok Thailand

**Keywords:** *Aonyx cinereus*, distribution, Laos, *Lutra lutra*, mtDNA diversity, Nakai‐Nam Theun

## Abstract

Otter populations are threatened by habitat loss, pollution, conflicts with humans, and illegal wildlife trade to meet the demand for pets, for their fur, and for parts used in traditional medicines. Baseline information on the distribution, population genetic diversity, and connectivity is crucial to inform conservation management decisions; however, reliable data from otter populations in Southeast Asia remain scarce. In this study, we conducted baseline otter fecal DNA surveys based on mitochondrial DNA (mtDNA) to identify species, assess the occurrence, and map the spatial distribution of genetic diversity and evolutionary relationships of otter populations using 1700 bp *Cytochrome B ‐ Control Region* and mitogenome from Nakai‐Nam Theun National Park in the Annamite Mountains of Lao PDR. Of the total 56 samples identified to species, the majority (87.5%) was of the widely distributed Eurasian otter with three haplotypes (*Lutra lutra*; LLLA01–LLLA03), with a calculated haplotype diversity of 0.600 and a nucleotide diversity of 0.00141 based on mitogenome. The second species was the Asian small‐clawed otter with only one haplotype detected (*Aonyx cinereus*; ACLA01). All Eurasian otter haplotypes were newly characterized and clustered within the strongly supported South–Southeast–North Asian clade of *Lutra lutra*. Compared with the European clade, the high mtDNA diversity of *Lutra lutra* in Nakai‐Nam Theun National Park potentially reflects long‐term demographic stability and lesser degree of population bottleneck during the last glacial maxima (LGM, ~21,000 years ago). The single haplotype detected in Asian small‐clawed otters had not been detected in previous genetic studies. Our research is the first otter‐specific noninvasive genetic study in Lao PDR and provides baseline insights into the otter population diversity in a regional priority site for biodiversity conservation.

## Graphical abstract

We conducted baseline fecal DNA surveys based on mitochondrial DNA (mtDNA) to identify otter species, assess their occurrence, and map the spatial distribution of their genetic diversity, in Nakai‐Nam Theun National Park in the Annamite Mountains of Lao PDR. We confirm the occurrence of two otter species: Eurasian otter *Lutra lutra* and Asian small‐clawed otter *Aonyx cinereus*. The haplotypes (3 for *L. lutra* and 1 for *A. cinereus*) were detected for the first time.

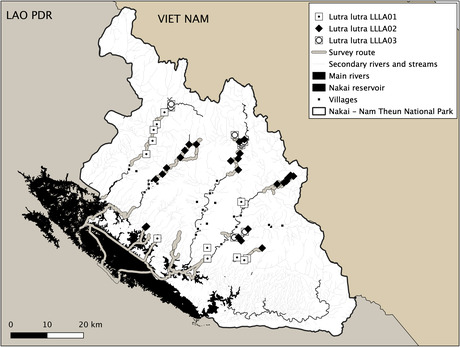

## 1 INTRODUCTION

Of the thirteen described otter species spread around the world, four range in Asia. Yet, their distribution and conservation status remain very little known or studied. The global conservation status of all four Asian otter species is marked by a declining population trend throughout their range (Duplaix & Savage, 2018), excepting the recovery of populations of *L. lutra* in some European countries (Duplaix & Savage, 2018; Roos et al., 2021). These declines are attributed to the continuous loss of habitat, decreasing number of their prey overharvested by humans, illegal hunting for wildlife trade, and illegal capture to supply exotic pet markets, particularly in Asia, where social media have driven the demand for keeping otters as pets (Gomez & Bouhuys, 2018; Harrington et al., 2019; Kitade & Naruse, 2018; McMillan et al., 2021; Siriwat & Nijman, 2018).

In Lao People's Democratic Republic (Lao PDR), three species have been confirmed to occur, listed globally as Near Threatened (NT) or Vulnerable (VU) on the IUCN Red List of Threatened Species: Asian small‐clawed otter *Aonyx cinereus* (VU), Smooth‐coated otter *Lutrogale perspicillata* (VU), and Eurasian otter *Lutra lutra* (NT) (Coudrat et al., 2014a; Dersu & Associates, 2008; Duckworth, 1997; Duckworth et al., 1999; Timmins & Evans, 1996). The latter has only been confirmed from one published historical record from Northern Lao PDR (Delacour, 1940 in Duckworth et al., 1999). There are no known records of the Hairy‐nosed otter *L. sumatrana* in Lao PDR, though the lack of otter surveys in the country means the presence of the species cannot be ruled out.

In Laos PDR, the three otter species included in the national Wildlife and Aquatic Law (*L. lutra, A. cinereus, L. perspicillata*) are listed in the *Prohibited Category I* (which designates species for which hunting is prohibited anywhere and at any time in the country) (MAF, 2007). The three species confirmed in Lao PDR are listed in Appendix I of the Convention on International Trade in Endangered Species of Wild Fauna and Flora (CITES, 2022). Despite the legal protection, otters have often been recorded in the trade in Lao PDR, sold for their skin (Nooren & Claridge, 2001).

Nakai‐Nam Theun National Park (NP) has been identified as an important area for the conservation of otters in Lao PDR and Southeast Asia (Duplaix & Savage, 2018; Timmins & Evans, 1996). In the past two decades, the NP has been under severe and increasing pressure from illegal wildlife hunting, mostly driven by the lucrative international wildlife trade (Coudrat et al., 2014a, 2014b; Nooren & Claridge, 2001; Robichaud et al., 2009). Otters have been targeted in Nakai‐Nam Theun NP by villagers in the past for illegal trade but at a fluctuating trend based on a regional trade demand. Otters are often reported destroying local villagers' fishnets; however, retaliatory killing in response to conflicts and subsistence hunting do not seem prevalent in the area (Coudrat, 2016). Nonetheless, the growing human population within the national park (over 8000 people, with an annual growth rate of 2.69%; DAFO, 2021), heavily reliant on fish and forest products for their livelihoods may increase human‐otter conflicts and pressure on otter populations in the near future.

There is an urgent need to monitor otter distribution and population status in Nakai‐Nam Theun NP; however, species identification of otters, population genetic diversity, and their adaptive potential have been uncertain. At least two species have been reported: *A. cinereus* and another larger otter species, unidentified species (see Dersu & Associates, 2008; Duckworth, 1997; Timmins & Evans, 1996). Otter footprints and spraints (otter's feces) have commonly been observed during surveys (e.g., Coudrat, 2016; Dersu & Associates, 2008; Timmins & Evans, 1996; Coudrat *pers. Obs*. 2022). In 2016, an otter‐specific camera‐trap survey along one of the main rivers only photographed *A. cinereus* (Coudrat, 2016). Identifying otter or other sympatric small carnivore species based solely on tracks, feces, direct sightings, or photographs (e.g., camera traps) can be challenging; ambiguous scat morphology, footprints, and photographs may lead to unresolved species identification (Akrim et al., 2018; Dersu & Associates, 2008; Timmins, 2018; Timmins & Evans, 1996). The paucity of surveys and records of confirmed otter species in Lao PDR hamper the understanding of their population status and ultimately the ability to adopt species‐specific and site‐based management and conservation strategies.

Noninvasive fecal sampling and population genetic analyses have been increasingly applied to overcome limitations from observation‐based species identification. Mitochondrial DNA (mtDNA) diversity based on *Cytochrome B* gene (Koepfli et al., 2008; Moretti et al., 2017) and partial *Control Region* (Ferrando et al., 2004; Finnegan & Néil, 2010; Hwang & Cho, 2018; Mucci et al., 2010; Pérez‐Haro et al., 2005; Stanton et al., 2009) has been used to map otter distribution, assess population connectivity and evolutionary history. Analyses using the complete mtDNA, or mitogenome, increase the number of parsimony informative sites and provide high resolution to phylogenetic reconstruction (Kim & Jo, 2021; Salleh et al., 2017).

In this study, we conducted for the first time in Lao PDR an otter‐specific survey using fecal sampling and population genetics based on 3 datasets (*Cytochrome B*, *Control Region*, and mitogenome) to assess otter occurrence, mtDNA diversity, historical connectivity, and evolutionary relationships of otter populations in Nakai‐Nam Theun NP with other Asian and European populations. The results of this baseline study will help designing future research and developing site‐based conservation programs for otters.

## 2 MATERIAL AND METHODS

### 2.1 Study site

**FIGURE 1:**
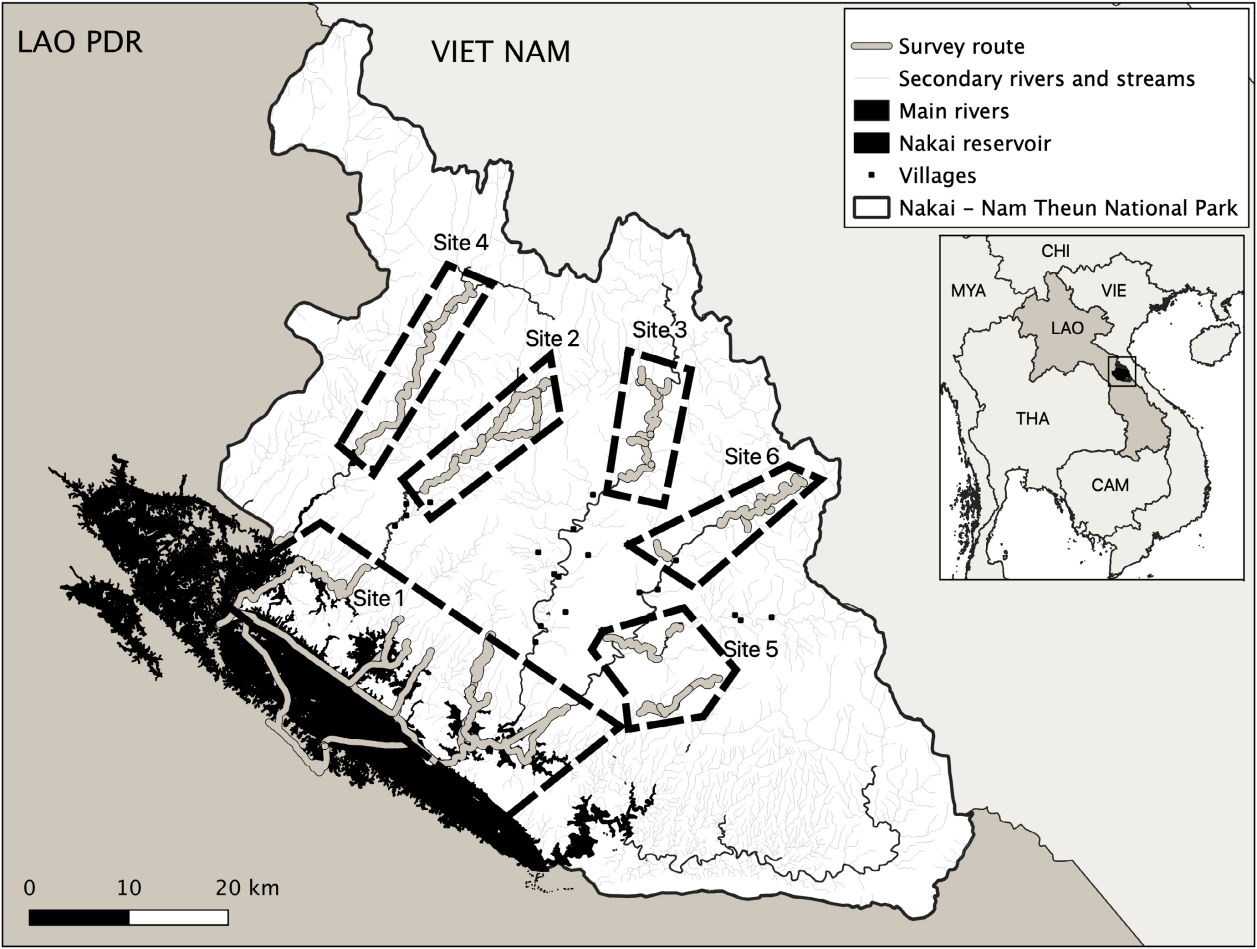
Nakai‐Nam Theun National Park (NP) in central Lao PDR and the six surveyed sites and survey routes in the NP in 2019–2020. Site 1: Nam Theun reservoir (Nov‐Dec 2019); site 2: Nam Mon‐Thongkhacheng (Dec 2019); site 3: Nam Theun (Jan 2020); site 4: Nam Xot (Feb 2020); site 5: Nam Mon‐Nam Pheo (Apr 2020); site 6: Nam Noy (May 2020).

Nakai‐Nam Theun NP (Figure 1) covers over 3500 km^2^ with altitudes ranging from 500 to >2200 m above sea level (asl). Around 80% of the protected area remains forested (Ferrand & Moore, 2018). The NP is dominated by old growth, largely undisturbed, dry‐evergreen forest, with other localized habitat types including pine, semi‐evergreen, upper‐montane, and wet‐evergreen forests (Timmins & Evans, 1996). In 2008, the western part of the park was inundated with a 450 km^2^ reservoir after the construction of the Nam Theun 2 hydroelectric dam. Five main rivers cross the NP, along which 13 village settlements occur (Figure 1). Nakai‐Nam Theun NP is ranked as a priority for its high biodiversity contribution at the national and global level (Coudrat, 2021); it falls in the heart of one of the richest regions of Southeast Asia in terms of biodiversity and endemism (Catullo et al., 2008) and is one of the “key biodiversity areas” within the Indo‐Burma biodiversity hotspot (Tordoff et al., 2012). This is notably due to the unique ecosystem characteristics of the Annamite mountains range, notably supporting a variety of large mammals (MacKinnon, 2000).

### 2.2 Field data collection

From December 2019 to May 2020, six sites were surveyed across Nakai‐Nam Theun NP (Figure 1) selected based on accessibility and substrate where otter feces could easily be observed and collected (i.e., larger rivers). Fecal samples were collected and placed in 50 ml sterile polypropylene centrifuge tubes containing 25 ml NaCl‐saturated DMSO buffer to ensure the stabilization of the DNA. Single‐use gloves and plastic spoons were used for each sample to avoid cross‐contamination between samples. We collected about 5 g from each sample, selecting the outside layer of the feces where gut epithelial cells are more abundant. Spraints with anal gland secretions were also targeted due to higher DNA concentration and amplification success (Sittenthaler et al., 2021).

For each sample, we recorded a unique identification code, date, time, GPS coordinates (in UTM WGS84 datum with a *Garmin GPSMap 64*), collector name, age (*fresh*: < 3 days old or *old*: > 3 days old), exposition (direct sunlight; rain; shade). During each field trip (10–15 days), the samples were kept away from direct sunlight. The samples were then kept in refrigerators (~4°C) for up to 15 days before they were transferred to the laboratory at the Faculty of Veterinary Medicine, Kasetsart University Kamphaeng Saen Campus, Nakhon Pathom Province, Thailand. Prior to the export of samples, an international certificate of compliance (reference number: Lao ABS‐CNP/Appl/RS001‐0220) to the Nagoya Protocol on *Access and Benefit‐sharing* was issued by the Institute of Biotechnology and Ecology of the Ministry of Science and Technology of Lao PDR. All laboratory procedures were conducted in full compliance with the *Institutional Animal Care and Use Committee* of Kasetsart University (Approval ID# ACKU64‐VET‐057).

### 2.3 Laboratory methods

Genomic DNA was extracted using QIAmp DNA Stool Mini Kit (Qiagen, Germany), with the modification of the final AE buffer to 60 μl for the first and second elution. To reduce DNA shearing and achieve long‐amplicon amplification (1700 bp), we gently mixed reagents in the tubes by inversion and avoided vigorous vortexing. The lysis time was increased from 1 hr to an overnight incubation (8‐12 hr) to increase DNA yield and PCR success rate. Mitogenome primer set was designed using the reported mitogenome of Lutrinae (Table A1) and the ones used in this study are reported in Table A2. All primers were mtDNA specific, excluding co‐amplification of the nuclear copies of mtDNA (*numts*), which were previously reported to overestimate diversity matrices in mammals (Hazkani‐Covo et al., 2010; Song et al., 2008). To assign mtDNA haplotype to each sample, a 1700 bp fragment spanning the complete *Cytochrome B* (*CytB*, 1140 bp), tRNA‐Thr, tRNA‐Pro, and 388 bp *Control Region* (*CR*) was amplified using the developed primer set: L14120 and LcanR7. For samples that failed to amplify the long fragment, two shorter fragments of 947 bp sequence (primer L14120/L15000R, 5′ ‐GAGGTGTGTAGTAGTGGGACG ‐ 3′) and 760 bp sequence (primer HKKlutFw 5′ ‐ CCCTAATCTTATCCATCCTAATCC ‐ 3′/LcanR7) were amplified and combined to obtain a 1700 bp sequence. Each 30 μl PCR reaction was optimized for degraded DNA by using nested PCR of the same primer pair and adding Bovine Serum Albumin (BSA) to reduce inhibition of PCR: 1 μl of DNA template, 5 μl of 2X Phire Tissue Direct PCR Master Mix (Thermo Scientific™, USA), 0.5 μM each of primer and reverse primers, 1 μl of 0.4% (w/v) BSA and 2 μl of sterilized double‐distilled water (ddH_2_O). Amplifications were performed using the following touch‐down profile: an initial denaturation of 98°C for 3 minutes, followed by 12 cycles of 98°C for 5 s, annealing at 66°C to 56°C (decreasing 2°C per 2 cycles) for 5 s and extension at 72°C for 30 s, and then 33 cycles with the same conditions but with the fixed annealing temperature at 56°C or 58°C (Table A2). PCR reactions were visualized on 1.5% agarose gel during electrophoresis, purified using FavorPrep™ Gel/PCR Purification Kit (Favorgen, Taiwan), and sent for sequencing at 1st BASE Laboratory, Malaysia. Four representative fecal samples from each haplotype were selected for mitogenome sequencing of LLLA01, LLLA02, LLLA03, and ACLA01 using the same PCR profiles as L14120/LcanR7 primers with varying annealing temperatures shown in Table A2.

### 2.4 Phylogenetic and diversity analyses

Chromatograms were visually inspected and trimmed using Bioedit (Hall, 1999). Multiple sequences were aligned, assembled, and mapped to the reported mitogenome of *Lutra lutra* (GenBank Accession number LC049377; Waku et al., 2016) using Unipro UGENE software (Okonechnikov et al., 2012). Mitogenome sequences from this study and previous studies (Kim & Jo, 2021; Waku et al., 2016) were aligned using MAFFT web version (Katoh et al., 2019). Mitogenome annotation was performed using MITOS2 (Donath et al., 2019) and verified using BLAST in the NCBI web server. We subsequently checked for the start and stop codons of all 13 protein‐coding genes. To reconstruct phylogenetic relationships among mtDNA haplotypes of otters from this study and the reported CytB, CR, and mitogenome deposited in the NCBI database (https://www.ncbi.nlm.nih.gov/), we used three datasets: (1) the 1140 bp *CytB* sequences, (2) the 750 bp *CR* sequences, and (3) the mitogenome sequences with the exclusion of an entire *CR*. Two mitogenomes from the Family Mustelidae were used as outgroups: *Hydrictis maculicollis* (NC046485; Madisha et al., 2019), and *Enhydra lutris* (NC009692; Yonezawa et al., 2007). The best‐fitted nucleotide substitution model was selected based on Akaike's Information Criterion (AIC), corrected AIC (AICc), or Bayesian Information Criterion (BIC); bootstrap values and maximum likelihood phylogenetic analysis were performed in IQ‐TREE web server (http://iqtree.cibiv.univie.ac.at/; Trifinopoulos et al., 2016) using ModelFinder and ultrafast 10,000 bootstrap replicates (Hoang et al., 2018). The best‐supported nucleotide substitution model for mitogenome was GTR with gamma‐distributed rate heterogeneity (Γ). Trees were visualized and edited with drawn FigTree v.1.4.2 (Rambaut, 2016). High statistical support for each node was considered to be >85% bootstrap percentage and moderate supports ranged between 70 and 85% bootstrap values. We constructed Statistical Parsimony haplotype network estimations (TCS haplotype network) of *Lutra lutra* using PopART (Leigh & Bryant, 2015). By assuming the number of samples equal to the number of individuals, we calculated haplotype diversity (Hd) and nucleotide diversity (π) of *Lutra lutra* population using mitogenome data in DnaSP v. 6.0 (Rozas et al., 2017).

## 3 RESULTS

**FIGURE 2:**
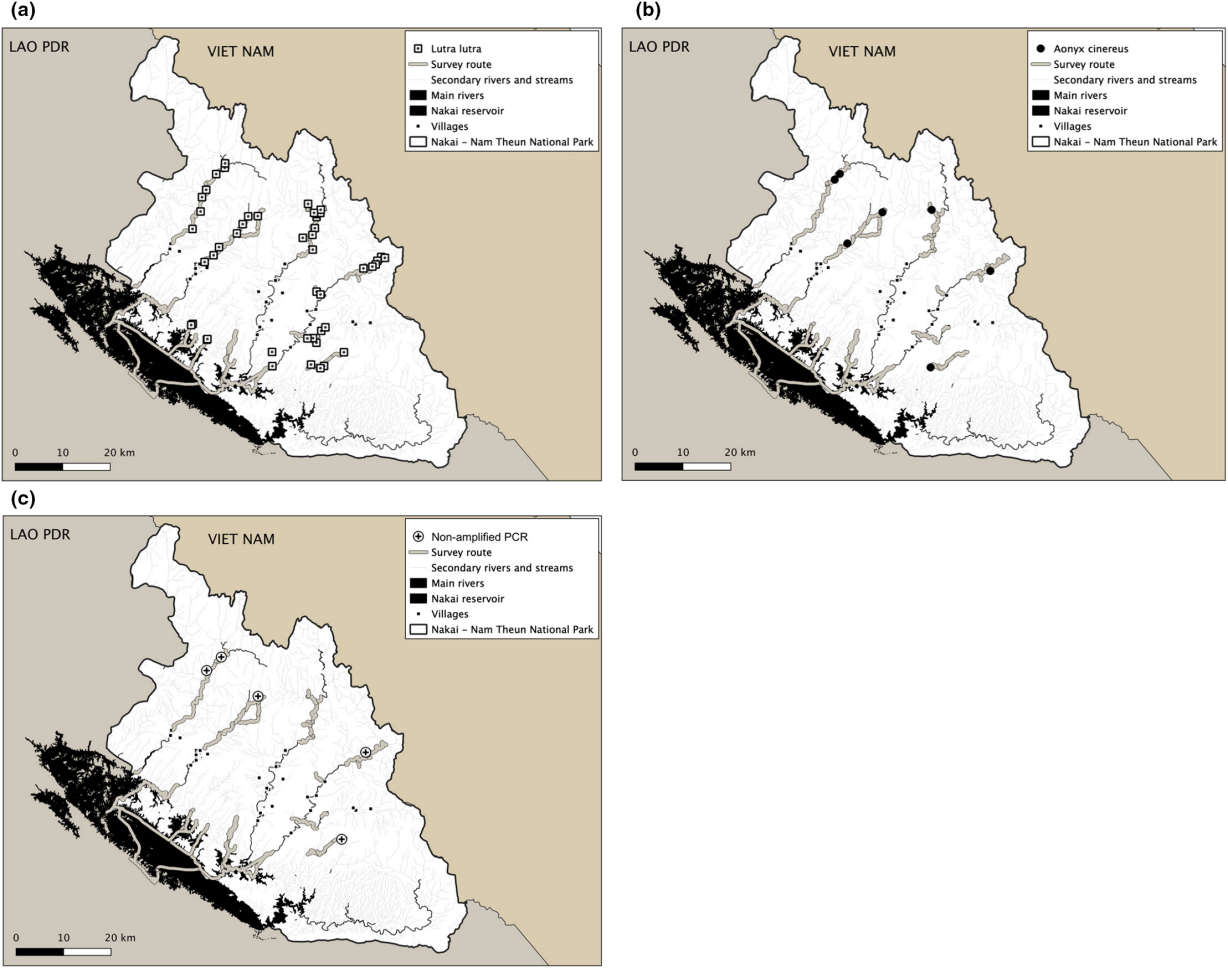
Distribution of otter species (*Lutra lutra* [a]; *Aonyx cinereus* [b]) and nonamplified PCR samples [c] from DNA extracted from 61 otter fecal samples collected in Nakai‐Nam Theun National Park in 2019–2020

Of the total 61 fecal samples (Table 1), we successfully amplified 56 samples (92% PCR success) using 3 primer pairs targeting 1700 bp sequences of *Cytochrome B* (CytB) and *Control Region* (CR) on mitogenome (Table A2). The high PCR success rate could be due to longer lysis incubation time (8‐12 hr) compared to previous studies (Hwang & Cho, 2018; Mucci et al., 2010). For the five samples for which amplification failed the targeted region was not amplified despite the use of the shortest fragment (<400 bp); we excluded them from further analyses. Of the 56 samples, we identified the majority (49 samples, 87.5%) as Eurasian otters (*Lutra lutra*), whereas the remaining samples were Asian small‐clawed otters (*Aonyx cinereus*) (Figure 2). We did not detect Smooth‐coated otter (*Lutrogale perspicillata*) in the samples we collected.

**TABLE 1:** Otter spraint samples collected at each of the six survey sites in Nakai‐Nam Theun National Park during 2019–2020 and otter species identified from DNA extraction from each sample.

Site name	Survey dates	Number of samples	*Lutra lutra*	*Aonyx cinereus*	Nonamplified PCR samples
Site 1: Nam Theun reservoir	29 Nov–5 Dec 2019	7	7	0	0
Site 2: Nam Mon‐Thongkhacheng	13–24 Dec 2019	10	7	2	1
Site 3: Nam Theun	10–17 Jan 2020	11	10	1	0
Site 4: Nam Xot	20–25 Feb 2020	11	7	2	2
Site 5: Nam Mon‐Nam Pheo	29 Mar ‐ 7 Apr 2020	11	9	1	1
Site 6: Nam Noy	21–27 May 2020	11	9	1	1
	Total	61	49	7	5

*Note*: *L. lutra* haplotypes = 3 (LLLAO01; LLLAO02; LLLAO03); *A. cinereus* haplotype = 1 (ACLAO01).

**FIGURE 3:**
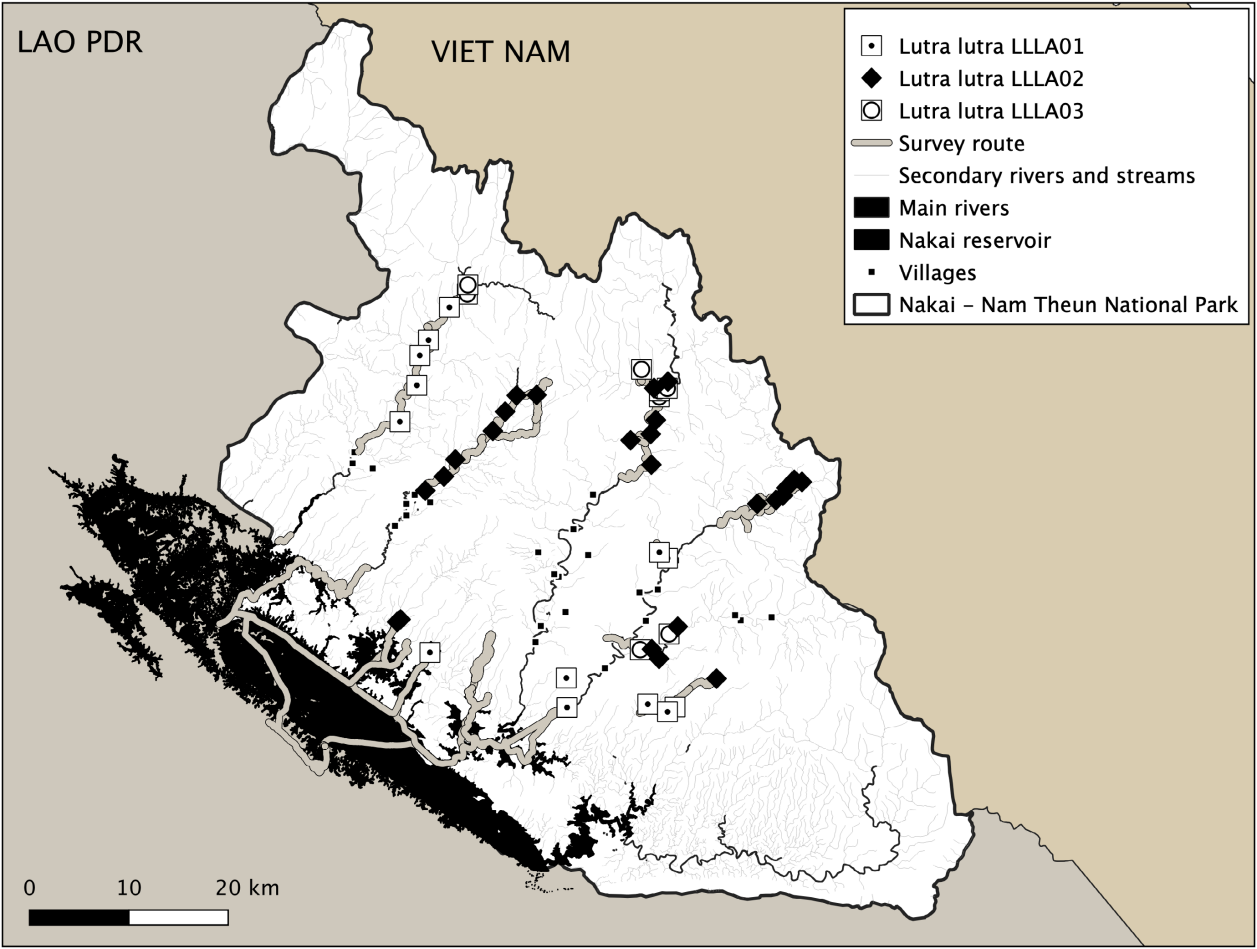
Distribution of the three *Lutra lutra* haplotypes identified from DNA extracted from 49 *Lutra lutra* fecal samples collected in Nakai‐Nam Theun National Park in 2019–2020

The 10 variable sites from 1700 bp sequences spanning the complete *Cytochrome B* (8 variable sites) until 388 bp *Control Region* (2 variable sites) defined 3 haplotypes (Table A3) in Eurasian otters: LLLA01, LLLA02, and LLLA03 and a single haplotype of Asian small‐clawed otters (ACLA01). The Eurasian otter haplotypes were newly characterized, and their spatial distribution was widespread across Nakai‐Nam Theun NP (Figure 3). LLLA02 was the most prevalent haplotype (27 samples, 55%), followed by LLLA01 (14 samples, 29%) and LLLA03 (8 samples, 16%). Haplotype diversity (Hd ± SD) and nucleotide diversity (π ± SD) based on the mitogenome of *L. lutra* population were 0.600 ± 0.047 and 0.0041 ± 0.00007, respectively.

**FIGURE 4:**
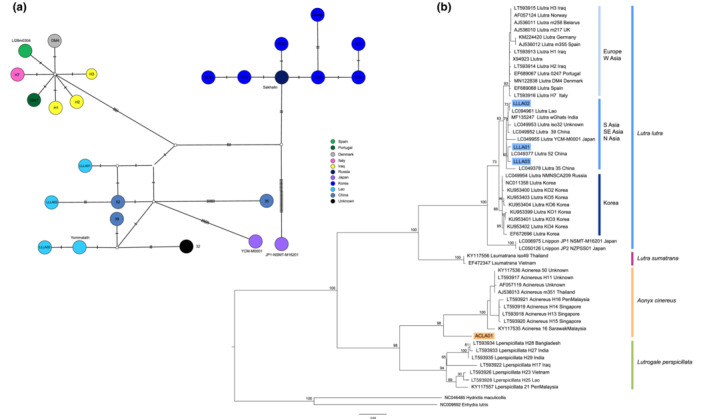
(a) TCS haplotype network of *Lutra lutra* mtDNA haplotypes based on 1140 bp *Cytochrome B* and maximum likelihood. Dash lines denote the number of mutations and the circle area is proportional to haplotype frequency. (b) Phylogenetic analysis of 1140 bp *Cytochrome B* Haplotypes from this study (*Lutra lutra*; LLLA01‐LLLA03, *Aonyx cinereus*; ACLA01) are highlighted in blue and orange, respectively. Branch lengths are proportional to the number of mutations, and numbers above branches represent bootstrap values in percentage.

Compared with LLLA02 haplotype, LLLA01 and LLLA03 haplotypes differed by 4 bp and were more closely related to each other. All three haplotypes were closely related to those reported from Lao and China (Waku et al., 2016) and clustered within the strongly supported Asian clade (South Asia, Southeast Asia, and North Asia) based on the TCS network and phylogenetic analyses of the complete *CytB* (Figure 4) and mitogenome (Figure A1), respectively. Due to the lack of variable sites within 388 bp *Control Region* sequences, LLLA01 and LLLA03 haplotypes could not be differentiated from each other (Figure A1), but 8 variable sites were detected within the complete 1140 bp *CytB* sequences (Table A3). With a lower number of parsimony informative sites within *Control Region*‐based phylogeny, statistical support for the European clade and Asian clade was lower (<70% bootstrap values, Figure A1) than *CytB*‐based phylogeny. Due to the limited number of mitogenome sequences from European and other Asian countries, mitogenome phylogenetic analysis also did not detect genetic partitioning into Asian and European regions (Figure A2). However, two clades corresponding to Lao‐China‐Japan groups (95% bootstrap values) and Korea‐Russia groups (91% bootstrap values) were strongly supported (Figure 4).

## 4 DISCUSSION

Our study is the first otter‐specific fecal DNA survey conducted in Lao PDR representing wild populations. Fecal DNA‐based species identification overcomes the challenges in differentiating closely related otter species with similar diet and scat morphology and can provide insights into their diversity and distribution. Our otter‐specific survey design allowed us to easily detect otters from their characteristic spraints, compared with camera‐trap monitoring programs focusing on ground mammals often failing to detect any otters as most surveys are not otter‐specific and thus cameras are rarely set in otters' habitats such as riverine, inland and coastal wetlands (Chutipong et al., 2014). Based on the analysis of the fecal samples we collected, two otter species were detected (*L. lutra* with three different haplotypes and *A. cinereus* with a single haplotype).

### 4.1 Occurrence of sympatric Eurasian and Asian small‐clawed otters

We confirm for the first time the presence of Eurasian otter in Nakai‐Nam Theun NP. Our results also detected the already known to occur Asian small‐clawed otter. Previous track and sign surveys in Nakai‐Nam Theun NP only confirmed the presence of Asian small‐clawed otter and of at least one other unidentified otter species (see below; Dersu & Associates, 2008; Timmins & Evans, 1996). However, mtDNA markers have limitations in detecting hybridization (see below for a more comprehensive discussion) that requires nuclear DNA‐based assignment and database of the known allele frequency of both parental species.

#### 4.1.1 Asian small‐clawed otter *Aonyx cinereus*

The occurrence of this species has been confirmed from our surveys and previous signs, camera‐trap photographs, and sightings (Coudrat, 2016; Dersu & Associates, 2008; Timmins & Evans, 1996). The null genetic diversity disclosed in the Nakai‐Nam Theun population might be due to the small sample size. Although the species is widely distributed across Asia, the extent of habitat loss and illegal pet trade at both international and national levels may be greater for Asian small‐clawed otter, compared with Eurasian otter, and threatens their long‐term viability (Gomez & Bouhuys, 2018; Harrington et al., 2019; Kitade & Naruse, 2018; McMillan et al., 2021; Siriwat & Nijman, 2018). The ACLA01 haplotype was not detected in previous genetic studies in Asia (Moretti et al., 2017; Waku et al., 2016), although such surveys are still limited. Extensive geographic survey coverage across Southeast Asia is needed to confirm whether the single haplotype of *A. cinereus*, found in this study, is area‐specific to Nakai‐Nam Theun NP.

#### 4.1.2 Eurasian otter *Lutra lutra*

We confirm for the first time the occurrence of the Eurasian otter which is otherwise difficult to document using traditional survey methods (e.g., sign surveys). Based on previous otter signs including spraints and tracks, as well as villagers' reports, a “large otter species” have often been reported in Lao PDR, including in Nakai‐Nam Theun NP, without a clear species identification possible between Eurasian otter or Smooth‐coated otter (Coudrat, 2016; Dersu & Associates, 2008; Timmins, 2018). The species had not been detected in a previous small‐scale otter‐specific camera‐trap survey conducted in the park in 2015, likely due to the small sampling period and the selected settings and locations of the cameras (Coudrat, 2016). We found three haplotypes for Eurasian otter in Nakai‐Nam Theun NP, suggesting a relatively stable population in the past and long‐term genetic exchanges across the landscape compared with a single dominant haplotype (Lut01; Stanton et al., 2009) that has been so far detected across all European countries. Similarly, a higher level of mtDNA diversity of Eurasian otter in Ireland, compared to other European countries (Ferrando et al., 2004), suggested the more stable demographic history and/or several colonization events in the past (Finnegan & Néil, 2010). In other countries across Europe, however, the dominant Lut01 haplotype of Eurasian otters suggests a severe population bottleneck during Pleistocene glaciations, followed by expansion from only a few refugia to recolonize available habitat during the postglacial periods (Cassens et al., 2000; Mucci et al., 2010). In Nakai‐Nam Theun NP, a single dominant haplotype was not detected, suggesting that population bottlenecks in Southeast Asia were potentially not as severe as in the European region (Mucci et al., 2010; Stanton et al., 2009). Given that we could not differentiate two haplotypes of *L. lutra* using *CR* sequences, based on our better results with the *CytB*, we recommend using the latter to assess intraspecific genetic diversity within *L. lutra*, as also found by the previous phylogenetic study (Koepfli et al., 2008).

#### 4.1.3 Smooth‐coated otter *Lutrogale perspicillata*

While it had been suggested that Smooth‐coated otter could occur in Nakai‐Nam Theun NP (Coudrat, 2016; Dersu & Associates, 2008; Timmins & Evans, 1996), we did not detect the species from our samples. Within little human‐disturbed areas this species is found mainly in lowland (Khoo et al., 2021). Such habitats in Nakai‐Nam Theun NP have been heavily disturbed by the Nam Theun 2 hydroelectric dam. It is possible that the species did occur and is now locally extinct, or that it has never occurred in Nakai‐Nam Theun NP and surrounding areas, although more intensive fecal sampling should be conducted in lowland riverine areas connected to the reservoir to confirm the presence of this species in the future. In addition, it is important to point out that species identification based on mtDNA presents some caveats in certain cases. In areas where phylogenetically close species are sympatric, cases of introgression are possible, which can result in the detection of mtDNA from one otter other than the investigated species. This has been detected in wild populations in Singapore and in museum specimens originated from Laos and Indonesia where mtDNA of Asian small‐clawed otter was found in Smooth‐coated otter (Barbanera et al., 2016; Guerrini et al., 2020; Moretti et al., 2017).

Knowledge on the distribution, population genetic diversity, and connectivity of otter species remains scarce across their range in Southeast Asia. The lack of such baseline information has weakened national and regional conservation interventions. Our otter fecal survey provides the first baseline reference for the species presence and genetic diversity in Nakai‐Nam Theun NP, where such surveys should be repeated in the future over wider areas and habitats. This noninvasive technique should also be expanded to other sites in Lao PDR. Our study, combined with a previous camera‐trap otter survey (Coudrat, 2016), suggests that Nakai‐Nam Theun NP retains healthy populations of *L. lutra* and *A. cinereus* and be considered a priority site for regional otter conservation. In addition to maternal mtDNA diversity, biparental markers with high mutation rates like microsatellite loci or genome‐wide single nucleotide polymorphisms (SNPs) are needed to determine with a much higher level of reliability both spatial structure and genetic diversity of the study populations.

## AUTHOR CONTRIBUTIONS

**Camille NZ Coudrat:** Conceptualization (equal); funding acquisition (equal); investigation (equal); methodology (equal); project administration (lead); visualization (equal); writing – original draft (lead); writing – review and editing (equal). **Wanlop Chutipong:** Writing – review and editing (equal). **Manakorn Sukmak:** Formal analysis (equal); funding acquisition (equal). **Supaphen Sripiboon:** Investigation (equal). **Worata Klinsawat:** Conceptualization (equal); formal analysis (lead); funding acquisition (equal); investigation (equal); methodology (lead); visualization (equal); writing – review and editing (equal).

## CONFLICT OF INTEREST

The authors declare no conflict of interest.

## Supporting information


**Figure A1.** TCS haplotype network of *Lutra lutra* and phylogenetic analysis of otter mtDNA haplotypes based on 306 bp control region sequences and maximum likelihood. Haplotypes from this study (*Lutra lutra*; LLLA01‐LLLA03, *Aonyx cinereus*; ACLA01) are highlighted in blue and orange, respectively. Branch lengths are proportional to the number of mutations, and numbers above branches represent bootstrap values in percentage. In network analysis, dash lines denote the number of mutations, and the circle area is proportional to haplotype frequency.

**Figure A2.** Phylogenetic analysis of otter haplotypes based on mitogenome sequences and maximum likelihood. Haplotypes from this study (*Lutra lutra*; LLLA01‐LLLA03, *Aonyx cinereus*; ACLA01) are highlighted in blue and orange, respectively. Branch lengths are proportional to the number of mutations, and numbers above branches represent bootstrap values in percentage.

**Table A1.** MtDNA sequences retrieved from the NCBI database for phylogenetic analyses

**Table A2.** MtDNA primers developed in this study and used to amplify otter mitogenome

**Table A3.** MtDNA haplotypes and variable sites from 1700 bp of the complete Cytochrome B (1140 bp), tRNA‐Thr, tRNA‐Pro, and 5’ control region (388 bp)

**Table A4.** Database of the otter fecal samples collected in Nakai‐Nam Theun National Park during 2019–2020 including unique identification code, collection date, collection time, GPS coordinates (in UTM WGS84 datum with a Garmin GPSMap 64), and DNA‐based species identification (NA = nonamplified PCR)

## Data Availability

The complete mitogenome sequences were deposited in NCBI GenBank (https://www.ncbi.nlm.nih.gov/genbank/) with the accession numbers: OP554562 to OP554565.
