# Supplementary figures and images for: Taxonomic status of otter species in Nakai‐Nam Theun National Park, Lao PDR, based on DNA evidence
Source: Ecol Evol. 2022 Dec 21;12(12):e9601. doi: 10.1002/ece3.9601 (PMC9771668; doi:10.1002/ece3.9601)

(A)

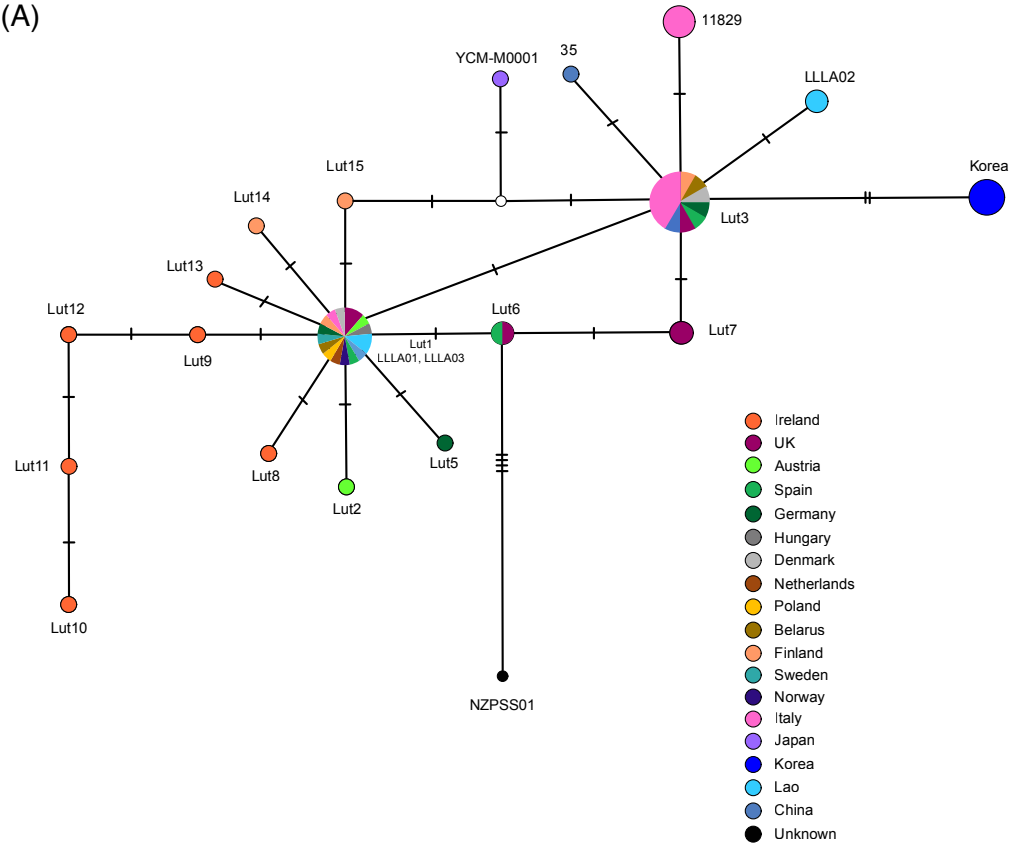

(B)

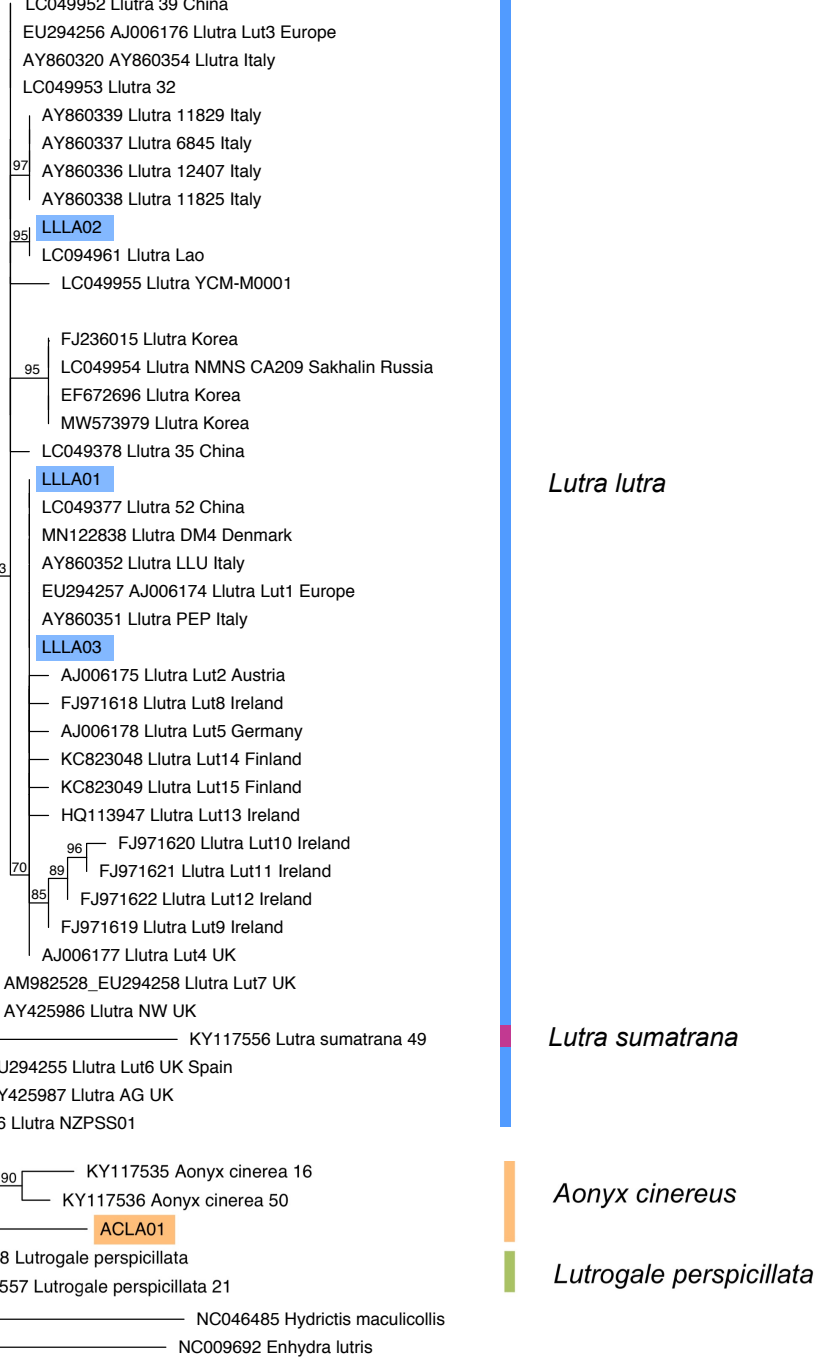

Supplement: Supplementary file 1 — Figure A1. TCS haplotype network of Lutra lutra and phylogenetic analysis of otter mtDNA haplotypes based on 306 bp control region sequences and maximum likelihood. Haplotypes from this study (Lutra lutra; LLLA01‐LLLA03, Aonyx cinereus; ACLA01) are highlighted in blue and orange, respectively. Branch lengths are proportional to the number of mutations, and numbers above branches represent bootstrap values in percentage. In network analysis, dash lines denote the number of mutations, and the circle area is proportional to haplotype frequency. [file ECE3-12-e9601-s002.pdf]

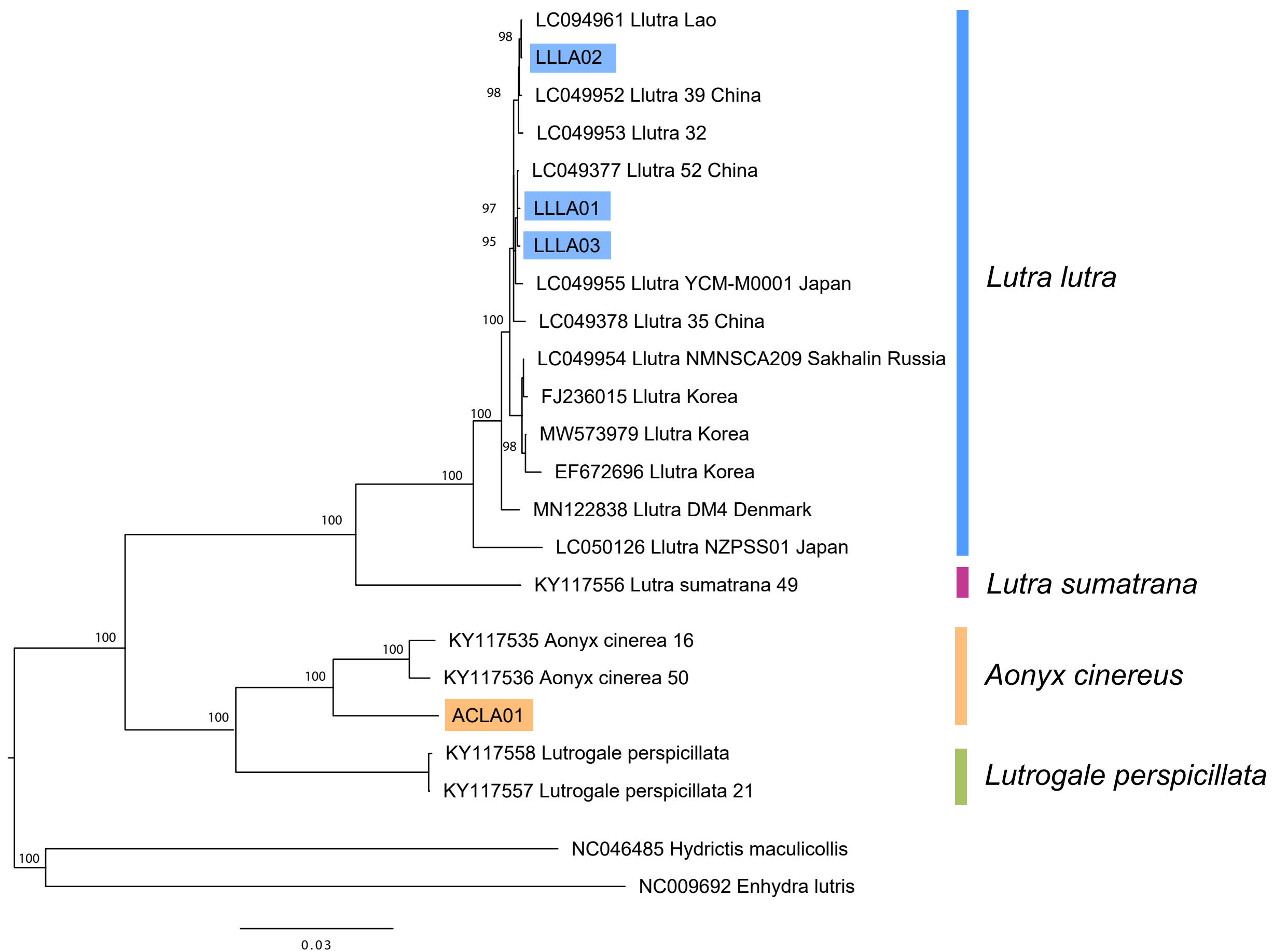

Supplement: Supplementary file 2 — Figure A2. Phylogenetic analysis of otter haplotypes based on mitogenome sequences and maximum likelihood. Haplotypes from this study (Lutra lutra; LLLA01‐LLLA03, Aonyx cinereus; ACLA01) are highlighted in blue and orange, respectively. Branch lengths are proportional to the number of mutations, and numbers above branches represent bootstrap values in percentage. [file ECE3-12-e9601-s003.pdf]
