# Supplementary material for: Taxonomic status of otter species in Nakai‐Nam Theun National Park, Lao PDR, based on DNA evidence
Source: Ecol Evol. 2022 Dec 21;12(12):e9601. doi: 10.1002/ece3.9601 (PMC9771668; doi:10.1002/ece3.9601)
Supplement: Supplementary file 3 — Table A1. MtDNA sequences retrieved from the NCBI database for phylogenetic analyses [file ECE3-12-e9601-s006.pdf]

No. / Species	GenBank Accession No.	Haplotype	Size length (bp)	Locality	Gene	Reference
Eurasian otter (*Lutra lutra*)						
1	LC049377	isolate 52	16492	China	mitogenome	Waku et al. 2016
2	LC049955	YCM:M0001	16536	Japan	mitogenome	Waku et al. 2016
3	LC094961	NA	16417	Lao (Khammouane, Yommalath)	mitogenome	Waku et al. 2016
4	LC049952	isolate 39	16537	China	mitogenome	Waku et al. 2016
5	LC049953	isolate 32	16536	Unknown	mitogenome	Waku et al. 2016
6	LC049378	isolate 35	16537	China	mitogenome	Waku et al. 2016
7	LC049954	NMNS:CA209	16536	Sakhalin, Russia	mitogenome	Waku et al. 2016
8	NC011358, FJ236015	NA	16536	Korea	mitogenome	Jang et al. 2009
9	MW573979	NA	16537	Korea	mitogenome	Kim and Jo 2021
10	EF672696	NA	16505	Korea	mitogenome	Ki et al. 2010
11	MN122838	DM4	16569	Denmark	mitogenome	DNAmark project, Margaryan, A. 2019. Natural History Museum of Denmark, Direct submission to GenBank
12	LC050126	NZP-SS-01	16519	Japan	mitogenome	Waku et al. 2016
13	LT593913	H1	1140	Iraq	*Cytochrome B*	Moretti et al. 2017
14	LT593914	H2	1140	Iraq	*Cytochrome B*	Moretti et al. 2017
15	LT593915	H3	1140	Iraq	*Cytochrome B*	Moretti et al. 2017
16	AF057124	NA	1140	Norway	*Cytochrome B*	Koepfli et al. 2008
17	AJ536011	isolate m258	402	Belarus	*Cytochrome B*	Marmi et al. 2004
18	AJ536010	isolate m217	402	UK	*Cytochrome B*	Marmi et al. 2004
19	AJ536012	isolate m355	402	Spain	*Cytochrome B*	Marmi et al. 2004
20	KM224420	FRT105	311	Germany	*Cytochrome B*	Naue et al. 2014
21	X94923	NA	1140	NA	*Cytochrome B*	Ledje and Arnason 1996
22	EF689067	isolate 02-0247	1140	Portugal	*Cytochrome B*	Fernandes et al. 2008
23	EF689068	isolate Ll28m0304	1140	Spain	*Cytochrome B*	Fernandes et al. 2008
25	LT593913	H1	1140	Iraq	*Cytochrome B*	Moretti et al. 2017
26	LT593914	H2	1140	Iraq	*Cytochrome B*	Moretti et al. 2017
27	LT593915	H3	1140	Iraq	*Cytochrome B*	Moretti et al. 2017
28	LT593916	H7	1140	Italy	*Cytochrome B*	Moretti et al. 2017
29	MF135247	NA	329	India	*Cytochrome B*	Madappa et al. 2018
31	KU953399	KO1	1140	Korea	*Cytochrome B*	Park et al. 2019
32	KU953400	KO2	1140	Korea	*Cytochrome B*	Park et al. 2019
33	KU953401	KO3	1140	Korea	*Cytochrome B*	Park et al. 2019
34	KU953402	KO4	1140	Korea	*Cytochrome B*	Park et al. 2019
35	KU953403	KO5	1140	Korea	*Cytochrome B*	Park et al. 2019
36	KU953404	KO6	1140	Korea	*Cytochrome B*	Park et al. 2019
37	LC006975	NSMT:M 16201	1272	Korea	*Cytochrome B*	Park et al. 2019
38	EU294257	Lut1	299	Europe	Control Region	Stanton et al. 2009
39	AJ006175	Lut2	299	Austria	Control Region	Cassens et al. 2000
40	EU294256	Lut3	299	Europe	Control Region	Stanton et al. 2009
41	AJ006177	Lut4	300	UK	Control Region	Cassens et al. 2000
42	AJ006178	Lut5	299	Germany	Control Region	Cassens et al. 2000
43	EU294255	Lut6	299	UK, Spain	Control Region	Stanton et al. 2009
44	EU294258	Lut7	299	UK	Control Region	Stanton et al. 2009
45	FJ971618	Lut8	254	Ireland	Control Region	Finnegan and Neill 2010
46	FJ971619	Lut9	254	Ireland	Control Region	Finnegan and Neill 2010
47	FJ971620	Lut10	254	Ireland	Control Region	Finnegan and Neill 2010
48	FJ971621	Lut11	254	Ireland	Control Region	Finnegan and Neill 2010
49	FJ971622	Lut12	254	Ireland	Control Region	Finnegan and Neill 2010
50	HQ113947	Lut13	344	Ireland	Control Region	Honnen et al. 2011
51	KC823048	Lut14	344	Finland	Control Region	Honnen et al. 2011

No. / Species	GenBank Accession No.	Haplotype	Size length (bp)	Locality	Gene	Reference
52	KC823049	Lut15	344	Finland	Control Region	Honnen et al. 2011
53	AY860320	NA	1116	Italy	Control Region	Ketmaier and Bernardini et al. 2005
54	AY860354	NA	918	Italy	Control Region	Ketmaier and Bernardini et al. 2005
55	AY860336	12407	241	Italy	Control Region	Ketmaier and Bernardini et al. 2005
56	AY860337	6845	241	Italy	Control Region	Ketmaier and Bernardini et al. 2005
57	AY860338	11825	241	Italy	Control Region	Ketmaier and Bernardini et al. 2005
58	AY860339	11829	241	Italy	Control Region	Ketmaier and Bernardini et al. 2005
59	AY860352	LLU	241	Italy	Control Region	Ketmaier and Bernardini et al. 2005
60	AY860351	PEP	241	Italy	Control Region	Ketmaier and Bernardini et al. 2005
61	AJ006174	Lut1	299	Europe	Control Region	Cassens et al. 2000
Hairy-nosed otter (*Lutra Sumatrana*)						
1	KY117556		16580	Bang Nara, southern Thailand	mitogenome	Salleh et al. 2017
2	EF472347		1140	Vietnam	*Cytochrome B*	Koepfli et al. 2008
Asian small-clawed otter (*Aonyx cinereus*)						
1	KY117536	isolate 50	16153	Unknown	mitogenome	Salleh et al. 2017
2	KY117535	isolate 16	16153	Sarawak, Malaysia	mitogenome	Salleh et al. 2017
3	LT593917	H11	1140	Unknown	*Cytochrome B*	Moretti et al. 2017
4	LT593918	H13	1140	Singapore	*Cytochrome B*	Moretti et al. 2017
5	LT593919	H14	1140	Singapore	*Cytochrome B*	Moretti et al. 2017
6	LT593920	H15	1140	Singapore	*Cytochrome B*	Moretti et al. 2017
7	LT593921	H16	1140	Peninsular Malaysia	*Cytochrome B*	Moretti et al. 2017
8	AF057119	NA	1140	NA	*Cytochrome B*	Koepfli et al. 2008
9	AJ536013	isolate m351	402	NA	*Cytochrome B*	Marmi et al. 2004
Smooth-coated otter (*Lutrogale perspicillata*)						
1	KY117557	Isolate 21	16042	Melaka, Malaysia	mitogenome	Salleh et al. 2017
2	KY117558	NA	16041	Bang Nara, southern Thailand	mitogenome	Salleh et al. 2017
3	LT593922	H17	1140	Iraq	*Cytochrome B*	Moretti et al. 2017
4	LT593926	H23	1140	Vietnam	*Cytochrome B*	Moretti et al. 2017
5	LT593927	H24	1140	Cambodia	*Cytochrome B*	Moretti et al. 2017
6	LT593933	H27	1140	India	*Cytochrome B*	Moretti et al. 2017
7	LT593934	H28	1140	Bangladesh	*Cytochrome B*	Moretti et al. 2017
8	LT593935	H29	1140	India	*Cytochrome B*	Moretti et al. 2017
Outgroup						
Aonyx capensis	MK309841	NA	16188	NA	mitogenome	Madisha et al. 2019
Hydrictis maculicollis	NC046485	NA	16308	NA	mitogenome	Madisha et al. 2019
Enhydra lutris	NC009692	NA	16431	NA	mitogenome	Yonezawa et al. 2007
