# Supplementary material for: Taxonomic status of otter species in Nakai‐Nam Theun National Park, Lao PDR, based on DNA evidence
Source: Ecol Evol. 2022 Dec 21;12(12):e9601. doi: 10.1002/ece3.9601 (PMC9771668; doi:10.1002/ece3.9601)
Supplement: Supplementary file 4 — Table A2. MtDNA primers developed in this study and used to amplify otter mitogenome [file ECE3-12-e9601-s001.pdf]

Pair	Mitochondrial Segments	Forward Primer	Forward Primer Sequence	Reverse Primer	Reverse Primer Sequence	Size (bp)	Ta (°C)
1	CytB - Control Region	L14120	CATGGAATCTAACCATGACTAGTGAC	LcanR7	CCAAATGCATGACACCACAGTTATGTG	1700	58
2	12S rRNA - 16S rRNA	L03F	GCACCCGGCTTACACCCAGG	L06R	CTGAACTCAGATCACGTAGGAC	1415	56
3	16S rRNA - ND1	L07F	CTAGGGATAACAGCGCAATCC	L09R	GCTGTTATGATGGGTAGGGCT	1280	56
4	ND1 - ND2	L10F	GAATCCGAGCATCCTATCCAC	L12R	TGAGGGGAATATGGTTAGTGC	1230	58
5	ND2 - COI	L13F	CCCCTCTCAGGATTTATCCC	L17R	AGCCCAGGAAGCCGATTGAT	1480	56
6	COI - COII	L16m7F	CATGCTACTCACAGACCGAAACCTG	L18m2R	GCGTCTATGGTGCTAGTATGCGTG	1220	56
7	COII - ATP8	L19F	GCGTACCCTCTCCAAATAGGCC	L23R	GCTGTTAGTCGTACGGCTAGGGC	1355	56
8	ATP8 - COIII	L23F	CTCTAGCCCACTTCTTACCACAAGG	L26R	GGGAGGATATCAGGTGGGATCGG	1625	56
9	ND3 - ND4L	L27F	CGCATTACTTCTACCACTACCRTGAG	L29R	GCCTAGTTTAAGGAGTACGGCGG	1165	56
10	ND4L - tRNA-His	L30F	TACCACTATACGGCCTTCAC	L32R	GGTCTGAGTGTAAGTACCACAT	1235	56
11	tRNA-His - ND5	L33F	CTGGCACTGGGTCACAATCC	L33R	GGTGGTGGGTGTAATGTTGTG	1200	56
12	ND5 - ND6	L36F	AACCCCGCTTCAACCCTATCA	L38R	GCTAATGGGGTGAGTTTTGCG	1125	58
13	Control Region - 12S rRNA	L44F	CGTGCATTAATGGTTTGCCCCATGC	L01aR	TCCTGGGTGTAAGCCGGGTG	1915	56
