# Supplementary material for: Taxonomic status of otter species in Nakai‐Nam Theun National Park, Lao PDR, based on DNA evidence
Source: Ecol Evol. 2022 Dec 21;12(12):e9601. doi: 10.1002/ece3.9601 (PMC9771668; doi:10.1002/ece3.9601)
Supplement: Supplementary file 5 — Table A3. MtDNA haplotypes and variable sites from 1700 bp of the complete Cytochrome B (1140 bp), tRNA‐Thr, tRNA‐Pro, and 5’ control region (388 bp) [file ECE3-12-e9601-s005.pdf]

mtDNA fragment		Cytochrome B								Control Region		
Haplotype	Sample Size	14295	14499	14595	14935	15060	15090	15183	15222	15684	15736	References
LC049377	-	A	C	A	C	A	A	T	T	T	A	Waku et al. 2016
LLLA01	14	•	•	•	•	C	•	C	•	•	•	This study
LLLA03	8	•	•	•	•	•	G	•	C	•	•	This study
LLLA02	27	G	T	G	T	•	•	•	•	C	G	This study
