# Supplementary material for: Taxonomic status of otter species in Nakai‐Nam Theun National Park, Lao PDR, based on DNA evidence
Source: Ecol Evol. 2022 Dec 21;12(12):e9601. doi: 10.1002/ece3.9601 (PMC9771668; doi:10.1002/ece3.9601)
Supplement: Supplementary file 6 — Table A4. Database of the otter fecal samples collected in Nakai‐Nam Theun National Park during 2019–2020 including unique identification code, collection date, collection time, GPS coordinates (in UTM WGS84 datum with a Garmin GPSMap 64), and DNA‐based species identification (NA = nonamplified PCR) [file ECE3-12-e9601-s004.pdf]

ID	Lab Sample ID	Field Sample ID	mtDNA haplotype	DNA-based Species	Date	Time	UTM_X	UTM_Y	Country	Site	Site_name
1	LNLA01	OT_NNT_SITE1_001	LLLA01	Lutra lutra	02 December 2019	10:49	540517	1964524	Laos	NKNT NP	reservoir
2	LNLA02	OT_NNT_SITE1_002	LLLA01	Lutra lutra	02 December 2019	11:06	540534	1964537	Laos	NKNT NP	reservoir
3	LNLA03	OT_NNT_SITE1_003	LLLA01	Lutra lutra	02 December 2019	12:15	540460	1967505	Laos	NKNT NP	reservoir
4	LNLA04	OT_NNT_SITE1_004	LLLA01	Lutra lutra	03 December 2019	7:59	526737	1970064	Laos	NKNT NP	reservoir
5	LNLA05	OT_NNT_SITE1_005	LLLA02	Lutra lutra	03 December 2019	8:34	526843	1970203	Laos	NKNT NP	reservoir
6	LNLA06	OT_NNT_SITE1_006	LLLA02	Lutra lutra	03 December 2019	12:45	523778	1973391	Laos	NKNT NP	reservoir
7	LNLA07	OT_NNT_SITE1_007	LLLA02	Lutra lutra	03 December 2019	13:12	523442	1973144	Laos	NKNT NP	reservoir
8	LNLA08	OT_NNT_SITE2_001	ACLA01	Aonyx cinereus	13 December 2019	14:13	530755	1990213	Laos	NKNT NP	Nam Mon/Thongkhacheng
9	LNLA09	OT_NNT_SITE2_002	ACLA01	Aonyx cinereus	15 December 2019	9:12	538109	1996739	Laos	NKNT NP	Nam Mon/Thongkhacheng
10	LNLA10	OT_NNT_SITE2_003	NA	NA	16 December 2019	10:40	537397	1997353	Laos	NKNT NP	Nam Mon/Thongkhacheng
11	LNLA11	OT_NNT_SITE2_004	LLLA02	Lutra lutra	16 December 2019	8:40	537472	1995967	Laos	NKNT NP	Nam Mon/Thongkhacheng
12	LNLA12	OT_NNT_SITE2_005	LLLA02	Lutra lutra	17 December 2019	10:50	535455	1995918	Laos	NKNT NP	Nam Mon/Thongkhacheng
13	LNLA13	OT_NNT_SITE2_006	LLLA02	Lutra lutra	17 December 2019	14:45	534302	1994286	Laos	NKNT NP	Nam Mon/Thongkhacheng
14	LNLA14	OT_NNT_SITE2_007	LLLA02	Lutra lutra	18 December 2019	11:26	533068	1992347	Laos	NKNT NP	Nam Mon/Thongkhacheng
15	LNLA15	OT_NNT_SITE2_008	LLLA02	Lutra lutra	19 December 2019	15:44	529295	1989475	Laos	NKNT NP	Nam Mon/Thongkhacheng
16	LNLA16	OT_NNT_SITE2_009	LLLA02	Lutra lutra	20 December 2019	8:39	528147	1987769	Laos	NKNT NP	Nam Mon/Thongkhacheng
17	LNLA17	OT_NNT_SITE2_010	LLLA02	Lutra lutra	20 December 2019	9:55	526265	1986347	Laos	NKNT NP	Nam Mon/Thongkhacheng
18	LNLA18	OT_NNT_SITE3_001	LLLA02	Lutra lutra	12 January 2020	9:30	549461	1993448	Laos	NKNT NP	Nam Theun
19	LNLA19	OT_NNT_SITE3_002	LLLA03	Lutra lutra	13 January 2020	8:36	549816	1995777	Laos	NKNT NP	Nam Theun
20	LNLA20	OT_NNT_SITE3_003	LLLA03	Lutra lutra	13 January 2020	10:04	549826	1996376	Laos	NKNT NP	Nam Theun
21	LNLA21	OT_NNT_SITE3_004	LLLA03	Lutra lutra	13 January 2020	11:26	550616	1996633	Laos	NKNT NP	Nam Theun
22	LNLA22	OT_NNT_SITE3_005	LLLA02	Lutra lutra	13 January 2020	12:35	550683	1997306	Laos	NKNT NP	Nam Theun
23	LNLA23	OT_NNT_SITE3_006	LLLA02	Lutra lutra	14 January 2020	9:18	549287	1996648	Laos	NKNT NP	Nam Theun
24	LNLA24	OT_NNT_SITE3_007	ACLA01	Aonyx cinereus	14 January 2020	10:37	548383	1997257	Laos	NKNT NP	Nam Theun
25	LNLA25	OT_NNT_SITE3_008	LLLA03	Lutra lutra	14 January 2020	13:09	548031	1998540	Laos	NKNT NP	Nam Theun
26	LNLA26	OT_NNT_SITE3_009	LLLA02	Lutra lutra	15 January 2020	12:50	548947	1992033	Laos	NKNT NP	Nam Theun
27	LNLA27	OT_NNT_SITE3_010	LLLA02	Lutra lutra	16 January 2020	7:46	546924	1991410	Laos	NKNT NP	Nam Theun
28	LNLA28	OT_NNT_SITE3_011	LLLA02	Lutra lutra	16 January 2020	10:02	549016	1988951	Laos	NKNT NP	Nam Theun
29	LNLA29	OT_NNT_SITE4_001	LLLA01	Lutra lutra	22 January 2020	13:04	528694	2004780	Laos	NKNT NP	Nam Xot
30	LNLA30	OT_NNT_SITE4_002	ACLA01	Aonyx cinereus	22 January 2020	13:54	529178	2004798	Laos	NKNT NP	Nam Xot
31	LNLA31	OT_NNT_SITE4_003	NA	NA	23 January 2020	8:50	529661	2005618	Laos	NKNT NP	Nam Xot
32	LNLA32	OT_NNT_SITE4_004	LLLA03	Lutra lutra	23 January 2020	10:19	530499	2006118	Laos	NKNT NP	Nam Xot
33	LNLA33	OT_NNT_SITE4_005	LLLA03	Lutra lutra	23 January 2020	12:07	530557	2007040	Laos	NKNT NP	Nam Xot
34	LNLA34	OT_NNT_SITE4_006	ACLA01	Aonyx cinereus	24 January 2020	9:27	528117	2003591	Laos	NKNT NP	Nam Xot
35	LNLA35	OT_NNT_SITE4_007	NA	NA	24 January 2020	12:26	526571	2002795	Laos	NKNT NP	Nam Xot
36	LNLA36	OT_NNT_SITE4_008	LLLA01	Lutra lutra	24 January 2020	13:07	526591	2001486	Laos	NKNT NP	Nam Xot
37	LNLA37	OT_NNT_SITE4_009	LLLA01	Lutra lutra	24 January 2020	13:43	525717	1999942	Laos	NKNT NP	Nam Xot
38	LNLA38	OT_NNT_SITE4_010	LLLA01	Lutra lutra	24 January 2020	14:31	525417	1996940	Laos	NKNT NP	Nam Xot
39	LNLA39	OT_NNT_SITE4_011	LLLA01	Lutra lutra	24 January 2020	14:28	523718	1993278	Laos	NKNT NP	Nam Xot
40	LNLA40	OT_NNT_SITE5_001	NA	NA	30 March 2020	9:34	555009	1967160	Laos	NKNT NP	Theung-Nam Mon/Nam Pheo
41	LNLA41	OT_NNT_SITE5_002	LLLA02	Lutra lutra	30 March 2020	11:09	555568	1967443	Laos	NKNT NP	Theung-Nam Mon/Nam Pheo
42	LNLA42	OT_NNT_SITE5_003	ACLA01	Aonyx cinereus	01 April 2020	10:18	548156	1964224	Laos	NKNT NP	Theung-Nam Mon/Nam Pheo
43	LNLA43	OT_NNT_SITE5_004	LLLA01	Lutra lutra	01 April 2020	11:05	548654	1964867	Laos	NKNT NP	Theung-Nam Mon/Nam Pheo
44	LNLA44	OT_NNT_SITE5_005	LLLA01	Lutra lutra	01 April 2020	14:50	551394	1964577	Laos	NKNT NP	Theung-Nam Mon/Nam Pheo
45	LNLA45	OT_NNT_SITE5_006	LLLA01	Lutra lutra	01 April 2020	15:54	550639	1964103	Laos	NKNT NP	Theung-Nam Mon/Nam Pheo
46	LNLA46	OT_NNT_SITE5_007	LLLA03	Lutra lutra	02 April 2020	12:13	550796	1971925	Laos	NKNT NP	Theung-Nam Mon/Nam Pheo
47	LNLA47	OT_NNT_SITE5_008	LLLA02	Lutra lutra	02 April 2020	12:33	551672	1972669	Laos	NKNT NP	Theung-Nam Mon/Nam Pheo
48	LNLA48	OT_NNT_SITE5_009	LLLA02	Lutra lutra	03 April 2020	11:10	549792	1969438	Laos	NKNT NP	Theung-Nam Mon/Nam Pheo
49	LNLA49	OT_NNT_SITE5_010	LLLA02	Lutra lutra	03 April 2020	11:52	549063	1970394	Laos	NKNT NP	Theung-Nam Mon/Nam Pheo
50	LNLA50	OT_NNT_SITE5_011	LLLA03	Lutra lutra	03 April 2020	12:13	547897	1970342	Laos	NKNT NP	Theung-Nam Mon/Nam Pheo
51	LNLA51	OT_NNT_SITE6_001	LLLA02	Lutra lutra	23 May 2020	9:16	563390	1987427	Laos	NKNT NP	Nam Noy
52	LNLA52	OT_NNT_SITE6_002	LLLA02	Lutra lutra	23 May 2020	11:00	564168	1987215	Laos	NKNT NP	Nam Noy
53	LNLA53	OT_NNT_SITE6_003	LLLA02	Lutra lutra	23 May 2020	12:37	562596	1986617	Laos	NKNT NP	Nam Noy
54	LNLA54	OT_NNT_SITE6_004	LLLA02	Lutra lutra	23 May 2020	13:07	562243	1985836	Laos	NKNT NP	Nam Noy
55	LNLA55	OT_NNT_SITE6_005	LLLA02	Lutra lutra	23 May 2020	13:30	561549	1985389	Laos	NKNT NP	Nam Noy
56	LNLA56	OT_NNT_SITE6_006	NA	NA	24 May 2020	9:10	560065	1985536	Laos	NKNT NP	Nam Noy
57	LNLA57	OT_NNT_SITE6_007	ACLA01	Aonyx cinereus	24 May 2020	12:22	560627	1984448	Laos	NKNT NP	Nam Noy
58	LNLA58	OT_NNT_SITE6_008	LLLA02	Lutra lutra	24 May 2020	13:36	559666	1985016	Laos	NKNT NP	Nam Noy
59	LNLA59	OT_NNT_SITE6_009	LLLA01	Lutra lutra	26 May 2020	12:53	550646	1979547	Laos	NKNT NP	Nam Noy
60	LNLA60	OT_NNT_SITE6_010	LLLA01	Lutra lutra	26 May 2020	14:05	549825	1980141	Laos	NKNT NP	Nam Noy
61	LNLA61	OT_NNT_SITE6_011	LLLA02	Lutra lutra	26 May 2020	12:52	550646	1979547	Laos	NKNT NP	Nam Noy
